# Awareness About Medication-Related Osteonecrosis of the Jaw Among Dental Professionals: A Multicentre Study

**DOI:** 10.3290/j.ohpd.a43361

**Published:** 2020-07-04

**Authors:** Vathsala Patil, Shruthi Acharya, Ravindranath Vineetha, Krithi Nikhil

**Affiliations:** a Assistant Professor, Department of OMR, Manipal College of Dental Sciences, Manipal, Manipal Academy of Higher Education, Manipal, Karnataka, India. Designed and performed the study and prepared the manuscript.; b Associate Professor, Department of OMR, Manipal College of Dental Sciences, Manipal, Manipal Academy of Higher Education, Manipal, Karnataka, India. Initiated the idea and hypothesis behind the study; assisted VP in performing the study and proofread the manuscript.; c Professor and Head, Department of OMR, Manipal College of Dental Sciences, Manipal, Manipal Academy of Higher Education, Manipal, Karnataka, India. Prepared the design and questionnaire for the survey.; d Consultant Statistician, Sattur, Dharwad, Karnataka, India. Performed statistical analysis of the obtained results.

**Keywords:** antiangiogenic drugs, antiresorptive agents, awareness, bisphosphonates, dentists

## Abstract

**Purpose:**

Bisphosphonates and non-bisphosphonate antiangiogenic and antiresorptive agents are widely used in the management of bone diseases and cancer. A subset of patients receiving these drugs can manifest with medication-related osteonecrosis of the jaw (MRONJ) and it is one of the major complications faced in dental practice. Dentoalveolar and periodontal surgery are the major risk factors associated with it. Therefore, a dentist must have adequate knowledge to promptly identify patients at risk and efficiently manage the condition. This multicentre study was designed with an aim to assess the level of knowledge and awareness regarding MRONJ among dentists from six dental schools.

**Methods and Materials:**

An online self-administered questionnaire was sent to all the dentists from six dental schools through Google forms. The results obtained were statistically analysed. The Kolmogorov–Smirnov test was performed to check for normality of data, while the Mann–Whitney U-test and chi-squared test were used to compare the responses to each question.

**Results:**

The questionnaire was sent to 570 dentists, out of which 234 responses were obtained. The majority of participants were aware of the term ‘MRONJ’ (83.3%), clinical indications of bisphosphonates (61.5%) and its mechanism of action (72.2%). However, 68.4% and 61.5% of dentists had no knowledge about the ‘drug holiday’ concept and risk factors associated with MRONJ, respectively.

**Conclusion:**

Although most of the participants had knowledge regarding certain aspects of MRONJ, such as mechanism of action and clinical indications of bisphosphonates, there was a lack of awareness about the drug holiday concept and drug-associated risk factors. This emphasises the need to spread awareness among the dental community, not only in tertiary healthcare centres, but also among private dentists and dental interns to prevent cases of MRONJ.

Bisphosphonates (BP) are synthetic analogues of pyrophosphate which act by antiosteoclastic and antiangiogenic activity, reducing bone turnover.^6^ Due to their strong bone-specific activity, they are widely used in the management of osteoporosis, in osteopenia to lessen bone fragility, to reduce the osteolytic lesions of multiple myeloma and in other bone disorders like Paget’s disease, osteogenesis imperfecta and bone metastasis in prostate, breast and lung cancers.^4^ They symptomatically benefit cancer patients by reducing bone pain, decreasing frequency of pathologic fractures, spinal cord compression and hypercalcemia of malignancy.^8^ Despite its positive impact on patients, a critical complication in subsets of patients receiving this drug has emerged, called osteonecrosis of the jaw (ONJ). Since 2003 numerous cases of osteonecrosis of the jaw in patients taking BPs have been reported in the literature and the American Association of Oral and Maxillofacial Surgeons (AAOMS) 2009 position paper has defined bisphosphonate-related osteonecrosis of the jaw (BRONJ) as ‘exposure of necrotic bone for more than 8 weeks in patients who are currently under BP or previous therapy of BP with no history of previous radiation therapy’.^10^ This nomenclature was changed to medication-related osteonecrosis of jaw (MRONJ) in 2014 to accommodate the growing number of osteonecrosis cases, with not only BPs but other non-bisphosphonate drugs like monoclonal antibodies and tyrosine kinase inhibitors which have antiresorptive and antiangiogenic action, such as denosumab and becacizumab.^12^ MRONJ is diagnosed when a patient on BP and/or non-BP antiresorptive, antiangiogenic agent with no previous history of radiation therapy presents with exposed bone in the maxilla or mandible for more than 8 weeks.^11^

Incidence of MRONJ is 10 patients/year/million populations, and with the increasing use of antiresorptive and antiangiogenic agents the prevalence is expected to rise.^13^ MRONJ with uncertain incidence and clinical features adversely affects the quality of life. Although numerous management recommendations have been suggested, since healing is non-achievable, prevention is the most effective way to limit its development. Dentoalveolar surgery and periodontal diseases are the main local risk factors, whereas the type of drug used, duration and route of administration are the medication-associated risk factors.^4^ Hence removal of all dental foci of infection prior to antiresorptive and antiangiogenic agent therapy commencement is absolutely essential. This requires the combined efforts of medical professionals who must promptly send every patient to be administered with bisphosphonates for dental evaluation and spread awareness of the side effects of these drugs among patients. Dentists should also promptly identify this group of patients as risk-prone and manage efficiently.^4^ There have been few studies about BRONJ awareness among medical professionals, oncologist and dentists in different regions but to the best of our knowledge only one study has assessed the awareness of MRONJ and results showed lack of awareness.^6,8,12^ With the number of patients on bisphosphonates and other antiresorptive agents on the rise, a good knowledge of the condition among dentists is an important determinant for prevention and management of MRONJ. Hence this multicentre survey was conducted to assess the level of knowledge and awareness regarding the MRONJ among dentists from all the dental schools in around our district.

## Materials and Methods

We conducted a multicentre study from May 2018 to November 2018, involving graduates, postgraduates and faculty from six dental schools (tertiary care centre). The postgraduates and faculty of the following dental specialties were included: Oral Medicine and Radiology, Oral Surgery, Periodontology, Endodontics, Prosthodontics and Implantology, as they are involved in diagnosis and management of patients on antiresorptive and antiangiogenic medications. The institutional ethical committee clearance was obtained for the same (IEC 98/2018).

A self-administered questionnaire was framed on the basis of information about MRONJ and its risk factors. The questionnaire was initially pilot tested in the same study setting for feasibility and interpretations were validated with 10 participants. Based on the feedback, the final questionnaire was drafted. The questionnaire consisted of three parts:

1. The first part of the questionnaire gathered information about the gender, age, specialty and years of experience;
2. The second part evaluated the participant’s knowledge about bisphosphonate class of drug, mechanism of action, clinical indications, route of administration and side effects;
3. The third part of questionnaire assessed participant’s awareness about the BRONJ, change of nomenclature to MRONJ, AAOMS guidelines, concept of drug holiday period, and risk factors for BRONJ.

The questionnaire was prepared on a Google form and the link was e-mailed to all participants. Information was gathered in an Excel spreadsheet and statistical tests were applied using SPSS 19.0 for Windows version (IBM SPSS Statistics software, Chicago, IL, USA)

### Statistical Analysis

The normality of the data was checked using Kolmogorov–Smirnov test which showed that data was not following normal distribution. Hence, a non-parametric Mann–Whitney U-test was performed to evaluate the difference in knowledge level among male and female. A descriptive analysis of responses to each question was also performed and answer of all the participants were analysed separately by means of chi-squared test for equal proportions. Thus, in each group analysed, a ‘p value’ was assigned in each question if frequency of an answer was statistically significant.

## Results

The questionnaire was sent to 570 dentists, out of which 234 responses were obtained. Out of 234 dentists, 176 were interns (n = 128), lecturers (17) and postgraduates (31) with fewer than 5 years of experience. Meanwhile, the 58 respondents with a postgraduate degree specialised in either Oral Medicine and Radiology (7), Oral Surgery (15), Periodontology (19), Endodontics (10), Prosthodontics or Implantology (7) and having more than 5 years of experience. The characteristics of the participants who responded to our survey is described in Table 1 and their response rate is described in Table 2. In our study 168 of dentists were females and 65 were males. There was no statistically significant difference in the overall knowledge level about MRONJ among males and females (Table 3).

**Table 1:** The details of participants who responded to the survey

		Frequency		Percent (%)
Gender	Males	65		27.8
	Females	168		71.8
Years of experience	Less than 5 years	Interns	128	75.2
		Postgraduate students	31	
		Lecturers	17	
		Total	176	
	More than 5 years	58		24.8

**Table 2:** The response rate of each group

	Total	Less than 5 years	More than 5 years
No. of dentists approached	570	330	240
No. of responses obtained	233	176	58
Response rate	40.8%	73.33%	33.5%

**Table 3:** The difference in knowledge level based on gender of participants

	Median scores (Q1, Q3)	U statistics	p value
Males	6 (4.8)	4919.5	0.70
Females	6 (5.8)		

A descriptive analysis of the responses to each question in the survey was made and results were compared with two different experience levels (less than 5 years and more than 5 years) as depicted in Table 4. Out of the 234 dentists who responded, 83.3% were aware of the recent nomenclature change of BRONJ to MRONJ, but only 35% could choose the correct definition of BRONJ. Although 61.5% and 72.2% of dentists were aware of the clinical indications for prescribing BPs and its mechanism of action, respectively, only 47.9% were aware of osteonecrosis as a potential side effect in patients on bisphosphonates undergoing dental surgery. About 72.2% could correctly identify the duration of BPs as one of the risk factors for developing MRONJ, only 31.6% were aware of the drug holiday concept and 34.6% were aware of AAOMS guidelines for the management of MRONJ.

**Table 4:** The distribution of the correct answers and missing responses to each question and association between years of experience

Questions asked	Total (234)	Participants who have not attempted the question	Less than 5 years (176)	More than 5 years (58)	p value
	% (n) of correct answers	% (n)	% (n) of correct answers	% (n) of correct answers	
1. Definition of BRONJ?	35.0 (82)	11.5 (27)	34.7 (61)	36.2 (21)	
2. What is MRONJ?	83.3 (195)	10.3 (24)	83.0 (146)	84.5 (49)	0.99
3. Clinical indications for prescribing bisphosphonates?	61.5 (144)	6.8 (16)	60.2 (106)	65.5 (38)	0.76
4. Mechanism of action of bisphosphonates?	72.2 (169)	8.5 (20)	68.8 (121)	82.8 (48)	0.74
5. Common route of administration of bisphosphonates?	54.7 (128)	8.1 (19)	53.4 (94)	58.6 (34)	0.15
6. Side effect of bisphosphonates in patients undergoing dental surgical procedure?	47.9 (112)	8.1 (19)	37.5 (66)	79.3 (46)	0.98
7. MRONJ commonly affects which jaw?	53.4 (125)	10.7 (25)	54.5 (96)	50.0 (29)	<0.001**
8. Which of the guidelines is followed for MRONJ?	34.6 (81)	18.4 (43)	33.0 (58)	39.7 (23)	0.62
9. Are you aware of the concept ‘drug holiday period’?	31.6 (74)	3.8 (9)	25.0 (44)	51.7 (30)	0.13
10. MRONJ has higher predilection in patient on parenteral (IV) antiresorptive agents than on oral supplements?	38.5 (90)	9 (21)	33.5 (59)	53.4 (31)	<0.001**
11. Risk of developing MRONJ is related to duration of intake of bisphosphonates?	72.2 (169)	11.5 (27)	67.6 (119)	86.2 (50)	0.004**

** indicates p value is statistically highly statistically significant.

## Discussion

Since the first report of BRONJ by Marx in 2003, it has generated greater interest in research among medical and dental professionals.^7^ The incidence of MRONJ on oral BPs is 0.001–0.1% and 1–12% in patients on intravenous BPs but with increasing use of antiresorptive, antiangiogenic agents, prevalence is expected to upsurge in the coming decades.^2,9^ The increase in cases of MRONJ has indicated an imminent need for dentists to have a broad and consolidate knowledge about the condition for prevention, early detection and effective management. Our study was performed with the aim to assess the knowledge and awareness of dentists about bisphosphonate therapy and MRONJ and its risk factors.

The response rate of our study was 40.8%, which was comparatively lower than previous studies performed on physicians and dentists.^1,8^ The majority of participants (83.3%) were aware of the change in nomenclature from BRONJ to MRONJ, however only 35% of the dentists could identify the correct definition of BRONJ. A better level knowledge was observed among specialised dentists with more than 5 years’ experience. This mixed response could be attributed to the fact that majority of our participants were new/recent graduates with less than 5 years of experience; hence their lack of exposure, inadequate experience and familiarity with the relevant literature could be the reason for incomplete knowledge.

The drugs causing the adverse effect of osteonecrosis of the jaw are bisphosphonates, and among non-bisphosphonates, antiresorptive agents and antiangiogenic agents. Familiarity with these drugs, their mechanism of action and clinical indications is required to promptly identify ‘at risk’ patients and take essential precautions during dental treatment.^5^ Bisphosphonates (BP) are highly bone-specific and alter bone turnover rate, hence are usually prescribed for the treatment of bone disorders. An antiresorptive agent like denosumab, which is a RANK ligand inhibitor and also acts similar to bisphosphonates by inhibiting osteoclastic function and bone resorption, is commonly prescribed to reduce fractures in osteoporotic patients to prevent skeletal events like metastatic bone disorders. However, in contrast to BP, the bone remodelling capacity of denosumab diminishes in 6 months after drug cessation. Antiangiogenic agents inhibit new blood vessel formation and are clinically indicated in gastrointestinal tumours, renal cell carcinomas and neuroendocrine tumours.^11^ A total of 72.2% of the dentists in our study knew about the mechanism of action of bisphosphonates and 61.5% could select the correct indications for prescribing bisphosphonates. This was similar to the findings by Kokane et al^5^ and our results were better than those obtained by de Lima et al.^6^ This factual knowledge will greatly reflect on the efficiency of the dentists involved in the management and prevention of MRONJ.

Our questionnaire also comprised of an open-ended question asking to list names of drugs which could cause osteonecrosis of jaw – only 14 dentists could recollect names like alendronic acid, zoledronic acid and pamidronate, with zoledronic acid being the most common answer. It was quite alarming to note that no other non-BP antiresorptive or antiangiogenic drugs were mentioned and the responses were similar to a previous study.^13^ It is of paramount importance for every dentist to be aware of all the names of causative drugs of MRONJ. During history recording, a patient might mention brand names instead of a drug name, and therefore a dentist might subsequently fail to identify the risk factor for MRONJ, thus jeopardising that patient’s care.

Type of drug, the duration of drug therapy, and route of administration are the drug-associated risk factors which influence the development of MRONJ. Patients on nitrogen-containing BPs, IV-administered BPs or non-BPs and longer duration of drug exposure have a higher risk for developing MRONJ.^13^ A knowledge of risk factors prepares the dentist to better deal with patient taking all precautionary measures during or prior to commencement of BP or any antiresorptive therapy. Although 72.2% of participants could identify the duration of drug therapy as a directly proportional risk factor, only 38.5% knew of the higher risks associated with administering antiresorptive or antiangiogenic agents through an IV route than when given orally. The concept of the drug holiday period defines temporary interruption of drug for prevention and management of MRONJ. Advocating a drug holiday for patients depends upon factors such as: the cumulative dose of drug, mode of drug intake and associated risk factors like systemic diseases, concomitant corticosteroid or antiresorptive agent intake. No drug alteration is suggested for patients on oral BP for less than 4 years with no clinical risk factors, discontinuation of drug is preferred for 2 months prior to and after surgery for patients on oral BP with risk factors and patients on intravenous BP.^11^ In our study, only 31.6% of participants were aware of this concept.

It is vital that along with dentists even the medical specialists who prescribe antiresorptive or antiangiogenic agents and patients who are taking these drugs should be aware of its adverse effects. Previous studies have shown <30% of medical doctors send these patients for dental referral prior to treatment.^4^ Usually, postmenopausal women presenting with osteoporosis are prescribed oral BP by general physicians, of which the patient is often unaware and may fail to report during dental examination.^3^ A thorough history taking, communication between dentists and medical specialists and providing detailed information about the adverse effects of MRONJ to patients is of utmost importance.

The limitations of this study include that the descriptive analysis of responses between dentists with less than and more than 5 years of experience was made, however a statistical analysis was not possible owing to unequal sample size in both groups. Also, since ours was a self-administered questionnaire, the response of the participants could be diluted due to informal discussions among the respondents contributing to response bias. Due to the high number of non-respondents and fewer numbers of senior faculty members participating in the study, it is possible that the study sample was not representative of target population, giving rise to selection bias. The participants in this group were from a tertiary healthcare sector, who had a better level of knowledge and experience due to continuous clinical exposure to cases of MRONJ. Hence their responses cannot be extrapolated to private dental practitioners.

## Conclusion

In this multicentre study, we evaluated the knowledge and awareness of MRONJ and its risk factors among dentists. Although most of the participants knew about the nomenclature change to MRONJ, mechanism of action and clinical indications of bisphosphonates, there was lack of explicit information about the drug holiday concept and drug-associated risk factors. This further emphasises the need to spread awareness not only among dentists in tertiary healthcare centres but also among young dental graduates and private dentists, through educational campaigns, lectures and workshops in collaboration with local dental associations, state and national bodies to strengthen their knowledge about MRONJ and prevent this morbid condition.
